# Phase 1 Study to Evaluate the Effect of the Investigational Anticancer Agent Sapanisertib on the QTc Interval in Patients With Advanced Solid Tumors

**DOI:** 10.1002/cpdd.808

**Published:** 2020-06-02

**Authors:** Chirag Patel, Sanjay Goel, Manish R. Patel, Lakshmi Rangachari, Jayson D. Wilbur, Yaping Shou, Karthik Venkatakrishnan, A. Craig Lockhart

**Affiliations:** ^1^ Millennium Pharmaceuticals Inc., a wholly owned subsidiary of Takeda Pharmaceutical Company Limited Cambridge Massachusetts USA; ^2^ Montefiore Medical Center Bronx New York USA; ^3^ Florida Cancer Specialists/Sarah Cannon Research Institute Sarasota Florida USA; ^4^ Metrum Research Group LCC Connecticut USA; ^5^ Sylvester Comprehensive Cancer Center Miller School of Medicine University of Miami Miami Florida USA

**Keywords:** anticancer drugs, arrhythmia, drug safety, modeling and simulation, pharmacokinetics

## Abstract

The aim of this phase 1 study was to determine the effects of sapanisertib on the heart rate–corrected QT (QTc) interval in patients with advanced solid tumors. Adult patients with advanced solid tumors were enrolled to receive a single sapanisertib 40‐mg dose. Blood samples for pharmacokinetic analysis were collected and electrocardiogram readings were recorded at baseline and up to 48 hours after dosing. Patients could continue to receive sapanisertib 30 mg once weekly in 28‐day cycles for up to 12 months. The primary objective was to characterize the effect of a single dose of sapanisertib (40 mg) on the QT interval. Secondary objectives were to evaluate safety, tolerability, and pharmacokinetics. Following a single sapanisertib 40‐mg dose in 44 patients, the maximum least squares mean (upper bound of 1‐sided 95% confidence interval) changes from time‐matched baseline were 7.1 milliseconds (11.4 milliseconds) for individual rate‐corrected QT interval at 24 hours after dosing, and 1.8 milliseconds (5.6 milliseconds) for Fridericia‐corrected QTc at 1 hour post‐dose. There was no sapanisertib plasma concentration‐dependent increase in the change from time‐matched baseline individual rate‐corrected QTc interval or Fridericia‐corrected QTc. The most common adverse events following sapanisertib 30 mg once‐weekly dosing were nausea (80%), fatigue (61%), vomiting (57%), and decreased appetite (45%). A single sapanisertib 40 mg dose did not produce clinically relevant effects on QTc interval in patients with advanced solid tumors. The safety profile of sapanisertib 30 mg once weekly was favorable, and no new safety signals were observed (NCT02197572, clinicaltrials.gov).

Assessment of the potential of an investigational agent to cause delayed ventricular repolarization as a biomarker for ventricular tachycardia is an essential component of new drug development. Drugs that cause delayed ventricular repolarization (QT interval/heart rate–corrected QT interval [QTc] prolongation) pose an increased risk for ventricular tachycardia and sudden cardiac death.^1^, ^2^ Prolongation of the QT interval associated with polymorphic ventricular tachycardia represents one of the more commonly reported toxicities that have resulted in the withdrawal or restricted use of postmarket approved drugs.^1^ In fact, drug‐related QTc prolongation resulted in the addition of black box warnings on 45 (8.2%) and the withdrawal of 16 (2.9%) of the 548 drugs approved by the US Food and Drug Administration between 1975 and 1999.^3^

Assessment of risk for QT/QTc prolongation is recommended for drugs in clinical development and, as per the International Conference on Harmonization (ICH) guidance, needs to be evaluated rigorously in a well‐controlled, thorough QT/QTc (TQT) study. TQT studies are typically conducted in healthy volunteers and include a placebo control and a positive control (ie, a drug known to prolong QT/QTc) to establish study sensitivity.^1^ This guidance also recommends using a higher dose (i.e., a supratherapeutic dose) of the investigational agent to assess the effects of higher drug concentration on QTc interval to exclude any drug effects on QTc that may result when drug concentrations are expected to be higher (eg, as a consequence of drug interactions or impaired organ function). However, the potential toxicity profile associated with anticancer agents often precludes their administration to healthy volunteers and presents unique challenges for conducting a TQT study. In cases where a TQT study cannot be conducted, a routinely used alternative approach is a dedicated QTc (DQT) study, representing a reduced study design, in which intensive electrocardiogram (ECG) data are collected prior to, and immediately following, dosing of the investigational agent.^4^, ^5^ These studies are typically designed such that ECG measurements coincide with clinically relevant maximal concentrations of the investigational agent and allow exposure‐response analysis to provide a robust assessment of the effect of the investigational agent on QTc interval. In addition, the E14 Working Group, which comprises industry and US Food and Drug Administration representatives, recommended a revision to the E14 guidelines to allow for concentration‐QTc modeling to be used as a primary analysis for assessing QTc prolongation risk. The group also published recommendations around study design and data analysis and interpretation to support regulatory submissions.^6^ Accordingly, the use of concentration‐QTc modeling as the primary analytical approach for evaluating the effects of investigational agents on the QT interval is now established and recognized in a Questions and Answers addendum to the ICH E14 guideline.^7^

Sapanisertib is a novel, selective, orally bioavailable inhibitor of mammalian target of rapamycin (mTOR)^8^, ^9^; the structure of sapanisertib has been published previously.^10^ In contrast to currently approved mTOR inhibitors, such as rapamycin and its analogues, that predominantly inhibit the mTORC1 complex,^11^ sapanisertib inhibits both mTORC1 and mTORC2 complexes and mitigates feedback activation of phosphoinositide 3‐kinase and AKT, which are known to cause resistance to mTORC1 inhibitors.^9^, ^12^ Sapanisertib is currently in phase 2 clinical investigation for the treatment of advanced or metastatic renal cell carcinoma (NCT03097328), breast cancer (NCT02756364), and endometrial cancer (NCT02725268), either as single agent or in combination with other anticancer agents. The recommended phase 2 dose of sapanisertib ranges from 4 mg once daily as a single agent (renal cancer) or once daily for 3 days each week in combination with paclitaxel (endometrial cancer) to 30 mg once weekly as a single agent (renal cancer) or in combination with fulvestrant (breast cancer).

Sapanisertib displays dose‐linear pharmacokinetics (PK) over the 2‐ to 40‐mg dose range.^13^ Absorption is fast following oral administration (median time to maximum plasma concentration, 0.5‐3.0 hours), and the terminal half‐life is 8 to 12 hours. Consistent with expectations from its single‐dose PK profile, sapanisertib displays minimal accumulation (accumulation ratio of ∼1) following once‐daily administration and does not accumulate following once‐weekly administration. In human hepatocytes, ∼60%, ∼20%, and ∼20% of the hepatic metabolism of sapanisertib was identified to be mediated by cytochrome P450 (CYP) isozymes, uridine 5'‐diphospho‐glucuronosyltransferases, and other non‐CYP enzymes, respectively (Takeda, data on file). In vitro reaction phenotyping studies suggest participation of CYP1A2, 3A4, and 2C19 in the oxidative metabolism of sapanisertib and uridine 5′‐diphospho‐glucuronosyltransferases 1A4, 2B10, and 1A3 in the conjugative metabolism of sapanisertib (Takeda, data on file). Sapanisertib did not inhibit or induce CYP enzymes at clinically relevant concentrations in vitro. While some inhibition of transport proteins has been observed in vitro (breast cancer‐ resistance protein half maximal inhibitory concentration [IC_50_], 51.9 mM; organic cation transporter 1 IC_50_, 18.9‐27.6 mM; organic cation transporter 2 IC_50_, 1.9 mM; Takeda data on file), plasma concentrations of sapanisertib at its highest clinical dose of 30 mg administered once weekly were not expected to reach concentrations that would inhibit these transporters. Taken together, the overall risk for drug‐drug interactions with sapanisertib as a potential victim or perpetrator of interactions is estimated to be low. No clinical PK drug‐drug interaction studies have been conducted with sapanisertib.

In vitro studies assessing the potential for sapanisertib to inhibit the human ether‐à‐go‐go channel shows that sapanisertib did not inhibit human ether‐à‐go‐go at concentrations well above the clinically anticipated concentrations. The IC_50_ value was 175 µM, which is ∼583 times the anticipated free maximum plasma concentration (C_max_) of 0.3 µM and ∼182 times the total C_max_ of 0.96 µM, at the highest dose of 40 mg sapanisertib that has been studied in cancer patients.^13^ An in vitro study also demonstrated that sapanisertib was modestly bound to human plasma proteins (70.5%; data on file). In addition, in a Good Laboratory Practice monkey telemetry study, there were no remarkable electrocardiographic changes after single‐ or multiple‐dose administrations (up to 0.5 mg/kg with estimated free C_max_ of 0.23 µM) of sapanisertib (data on file).

Here, we present the results of a DQT study of sapanisertib at a 40‐mg dose, the single‐agent maximum tolerated dose in the once‐weekly schedule, to determine the effects of sapanisertib on the QTc interval in patients with advanced solid tumors.

## Methods

### Study Design and Patients

This open‐label, single‐arm, phase 1 study was conducted in compliance with the institutional review board (IRB) regulations stated in Title 21 of the United States Code of Federal Regulations (US CFR), Part 56; the study protocol and other study‐related documents were approved by the following local or central IRB or independent ethics committees at all study sites: Biomedical Research Alliance of New York, LLC, Lake Success, New York; Washington University School of Medicine Human Research Protection Office, St. Louis, Missouri; IntegReview Ethical Review Board, Austin, Texas; and Mary Crowley Medical Research Center Institutional Review Board, Dallas, Texas. The study complied with the ethical principles of Declaration of Helsinki, the ICH Good Clinical Practice guidelines, all application local regulations, and the informed consent regulations stated in Title 21 of the US CFR, Part 50. All patients provided written informed consent.

The study was conducted at 5 centers across the United States (Montefiore Medical Center, Bronx, New York; Washington University School of Medicine, Saint Louis, Missouri; Stephenson Cancer Center, University of Oklahoma, Oklahoma City, Oklahoma; Mary Crowley Cancer Research Center, Dallas, Texas; and Florida Cancer Specialists/Sarah Cannon Research Institute, Sarasota, Florida) to evaluate the effect of a single oral dose of sapanisertib (40 mg) on the electrocardiographic QT/QTc interval in patients with advanced solid tumors (NCT02197572, clinicaltrials.gov). The primary end point was change from time‐matched baseline in the QTc interval (ΔQTc).

Patients reported to the study sites on day –1 for collection of serial baseline triplicate ECGs prior to sapanisertib administration (described in the ECG Assessments section). The patients reported to the sites fasted or having had a light meal that should have been completed at least 2 hours prior to the site visit. The patients were equipped with 12 ECG leads to continuously record patient ECG data onto a Holter H12+ ECG recorder. All procedures that were followed on day –1 were consistent with procedures expected for day 1, with the exception of plasma sample collection for PK assessments, since no sapanisertib was administered on day –1. After collection of the 10‐hour time point ECG on day –1, the patients were furloughed from the study site with Holter recorders still attached. These Holter recorders were to remain attached overnight to continue to collect ECG data into day 1.

On cycle 1 day 1, triplicate ECGs and plasma samples for PK were collected from patients ≤15 minutes prior to dosing and at 15 minutes; 30 minutes; and 1‐, 1.5‐, 2‐, 2.5‐, 3‐, 4‐, 6‐, 8‐, and 10‐hour time points after receiving a single oral dose of 40‐mg sapanisertib. The Holter recorders and ECG leads were removed from patients after collection of the 10‐hour time point ECG on day 1, following which patients were furloughed from site. Additional collections of ECGs and plasma samples for PK were conducted at 24 (cycle 1 day 2) and 48 hours (cycle 1 day 3) after dosing. After the first week, that is, cycle 1 day 8, patients had the option to continue to receive treatment with sapanisertib at a dose of 30 mg once weekly in continuous 28‐day cycles until disease progression, unacceptable toxicity, withdrawal of consent, or completion of the 12‐month maximum study duration.

Eligible patients were adults with a radiographically or clinically evaluable solid tumor. Full eligibility criteria are provided in the Supplemental Information.

### Electrocardiogram, PK, and Safety Assessments

To enable collection of time‐matched ECG and PK data before and after sapanisertib administration, patients reported to the study sites on day –1, during which ECG leads (12 leads) and Holter H12+ ECG recorders were attached. Each planned ECG collection time point was preceded by a 5‐minute supine rest period, after which triplicate ECGs were extracted from the Holter recorders at approximately 2‐ to 5‐minute intervals. Serial triplicate ECGs were collected from 0 to 10 hours, matched to the planned dosing on cycle 1 day 1 predose and postdose ECG and PK time points, to characterize the baseline ECG and QTc interval. All triplicate ECGs were extracted and read centrally (Biotelemetry, Malvern, Pennsylvania). All blood draws for PK were collected after the triplicate ECG collection period.

Plasma samples were analyzed for sapanisertib using a validated liquid chromatography with tandem mass spectrometry method with deuterated sapanisertib used as the internal standard. Multiple reactant monitoring was performed using a positive electrospray method for the mass transitions (m/z) of 310.0 to 268.1 for sapanisertib and 317.0 to 269.1 for deuterated sapanisertib. A Shimadzu high‐pressure liquid chromatography system and Sciex API 4000 tandem mass spectrometry system were used with an Agilent Eclipse plus C18, 50 × 4.6 mm, 3.50‐µm column. For extraction, 50 µL of diluent (0.1% formic acid in acetonitrile/water, 50:50, v/v) and 20 µL of internal standard solution (0.5 µg/mL in diluent) were added to 50 µL of ethylenediaminetetraacetic acid plasma followed by vortex mixing. Plasma proteins were precipitated by adding 500 µL of 0.2% formic acid in acetonitrile followed by vortex mixing for 2 minutes and centrifugation for 5 minutes. A 40‐µL aliquot of the supernatant was transferred to a high‐pressure liquid chromatography vial containing 400 µL of reconstitution solution (10 mM ammonium formate and 10 mM citric acid in acetonitrile/water, 20:80, v/v). Injection volume was 10 µL. A gradient mobile phase (from 20% to 98%) was used, composed of mobile phase A: 0.1% formic/4 mM ammonium formate in water and mobile phase B: 0.1% formic acid and 4 mM ammonium formate in acetonitrile/water 98:2, v/v). The sapanisertib retention time was 1.7 minutes. The dynamic range of the assay was 1 to 1000 ng/mL. Assay accuracy expressed as %bias for the quality control samples ranged from –4.0% to –2.7% and assay precision expressed as percentage coefficient of variation (%CV) for quality control samples, ranged from 3.0% to 9.5% in plasma.

PK parameters were calculated by noncompartmental methods using WinNonLin Professional version 6.1 (Certara, Princeton, New Jersey). The PK analysis population was defined as all patients who received at least 1 dose of sapanisertib and had sufficient concentration‐time data to calculate ≥1 PK parameters. The safety population included all patients who received ≥1 dose of sapanisertib, according to the National Cancer Institute Common Terminology Criteria for Adverse Events, version 4.03.

### Electrocardiogram Analysis and End Points

The baseline characterization of serial triplicate ECG allowed for patient‐specific individual heart rate correction (QTcI) in addition to the Fridericia's ($QTcF\;=\frac{QT}{\sqrt[{3}]{RR}}\;$) correction that is commonly used in clinical practice. The patient‐specific slopes were calculated using all pairs of QT and RR (time elapsing between two consecutive R waves in an ECG) interval data collected on day –1 and estimated using the linear regression expression:

$$\log\left(QT\right)=\log\left(a\right)+b*\log\left(RR\right)$$

to calculate the individual slope (b) for each patient. QTcI was then calculated as

$$QTcI=\frac{QT}{RR^{\left(b\right)}}\;$$

ECG analysis was conducted in the primary analysis population, which included patients who had all time points collected on Holter ECG monitoring on day –1 and day 1 of cycle 1, including ECG time points collected on cycle 1 day 1 to cycle 1 day 3 at 24 or 48 hours after dosing that had available baseline comparisons. Both QTcI and QTcF were found to be equally suitable as correction methods. There were no discernible differences in the QTcI‐RR relationships or QTcF‐RR relationships between baseline and postdose (Figure S1); however, the slope of the QTcI‐RR relationship was smaller (0.0068) than that of the QTcF‐RR relationship (0.0139), which supported the a priori designation of QTcI correction method as the primary method of analysis. Both ΔQTcI and ΔQTcF were calculated by subtracting the time‐matched day –1 (baseline) mean QTcI and QTcF values from the day 1 to day 3 mean QTcI and QTcF values, respectively.

For the categorical analyses, the categories used for frequency distribution (number and percentage of patients) for absolute QTcI and QTcF prolongation were: QTc intervals >450 milliseconds, >480 milliseconds, and >500 milliseconds, and those used for increase from baseline in QTc interval of >30 milliseconds and >60 milliseconds. Additional categorical analyses included the number and percentage of patients with QRS (interval on ECG between the start of the Q wave and end of the S wave) duration >110 milliseconds and 25% increase from baseline, and PR (interval on ECG between the start of the P wave and the beginning of the QRS complex) duration >200 milliseconds and 25% increase from baseline.

### Statistical Analyses

Based upon historical data, the intrapatient standard deviation in QTcF was assumed to be 9 milliseconds. A sample size of 30 evaluable patients would provide a half‐width of a 2‐sided 90% confidence interval (CI) for the mean change from baseline in QTcF of 2.7 milliseconds. To allow for 30 evaluable patients, 44 patients were enrolled in the study.

The primary analysis was a repeated‐measures mixed‐effects linear model that included nominal collection time as a fixed effect and the patient as a random effect. For each ECG parameter (ΔQTcI, ΔQTcF, heart rate, etc.), the point estimates of the least squares mean (LSM) changes from baseline at each time point and their 1‐sided upper 95%CI were estimated, which were used to make inferences of drug effect.

The relationships between concentration of sapanisertib and QTc and RR intervals were quantitatively analyzed in the PK/QTc analysis population using PK and ECG data from all patients. The data were first explored by graphical analysis, including a visual check for evidence of hysteresis. Mixed‐effects models were subsequently developed to describe the direct effect of sapanisertib on change from time‐matched baseline in RR, QTcI, and QTcF.

Standard diagnostic and goodness‐of‐fit plots were used for model evaluation and adequacy, plausibility, and precision of parameter estimates. A visual predictive check for the final model was generated to evaluate whether the model provided an accurate description of the data. The estimated parameters were reported with the standard error of the estimates as a measure of uncertainty, and nonparametric bootstrap resampling was used to construct 95%CI for the population parameters in each model. All model development was conducted with a qualified installation of the nonlinear mixed‐effects modeling software, version 7.3 (ICON Development Solutions, Hanover, Maryland). All data analyses were conducted using a qualified installation of the statistical software R.

## Results

### Patients

Overall, 44 patients with advanced solid tumors were enrolled and received a single dose of sapanisertib 40 mg on cycle 1 day 1. Of these, 42 (95%) patients had received prior antineoplastic therapy. A summary of patient baseline demographics and disease characteristics is shown in Table 1. Of the 44 patients, 32 patients completed all study‐specific assessments to provide sufficient data for the repeated‐measures mixed‐effects analysis of the effect of sapanisertib on QTc. Data from all 44 patients were included in the concentration‐QTc analyses.

**Table 1:** Patient Baseline Demographics and Disease Characteristics

	Total N = 44
Median age, y (range)	59.5 (22‐79)
Male, n (%)	16 (36)
Race, n (%)	
White	30 (68)
Black	9 (20)
Not reported	5 (11)
Ethnicity, n (%)	
Hispanic or Latino	7 (16)
Not Hispanic or Latino	29 (66)
Not reported	8 (18)
Median weight, kg (range)	70.6 (49‐122)
Disease type, n (%)	
Colon	5 (11)
Endometrial	5 (11)
Kidney	4 (9)
Colorectal	2 (5)
Sarcoma	2 (5)
Other^a^	26 (59)
Disease stage, n (%)	
<IV	6 (14)
IV	31 (70)
IVB	2 (5)
IVC	1 (2)
Not available	4 (9)
ECOG performance status	
0	13 (30)
1	30 (68)
2	1 (2)

ECOG, Eastern Cooperative Oncology Group.

a Includes anal, cervical, gastric, liver, melanoma, non–small cell lung cancer, ovarian, small cell lung cancer, soft tissue, stomach, thyroid, and “other.”

### Individual Rate‐Corrected QT Interval and QTcF Assessments (Repeated‐Measures Mixed‐Effects Analysis Population)

**Figure 1:**
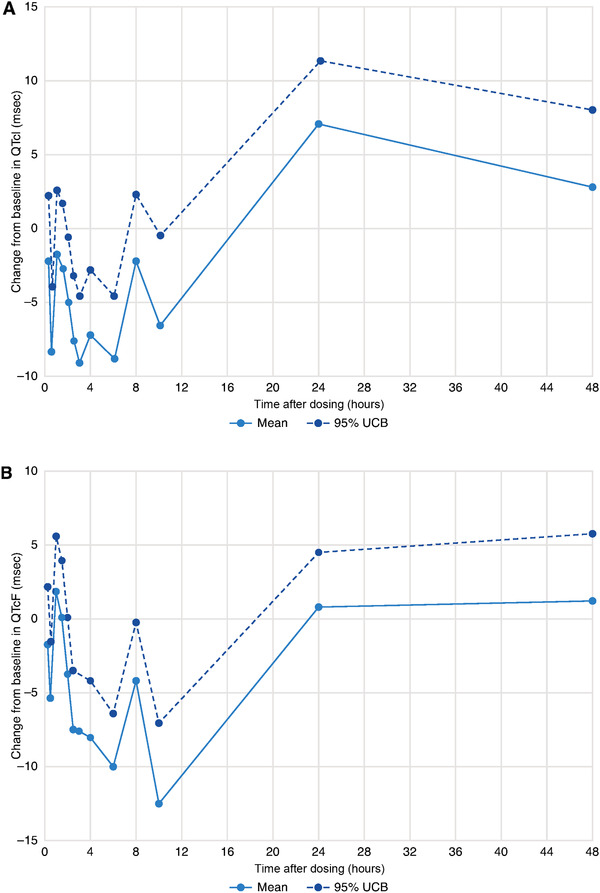
Mean time‐matched changes from baseline in (A) QTcI and (B) QTcF. QT, measure of the time between the start of the Q wave and the end of the T wave in the electrical cycle of the heart; QTcF, rate corrected QT interval with Fridericia correction; QTcI, individual baseline corrected rate‐corrected QT interval; UCB, upper confidence bound.

QTc data collected from patients during the PK/ECG analysis period (cycle 1 day 1 to day 3) showed that at time‐matched baseline (day –1), the overall mean QTcI values were normal, from 411.7 to 422.1 milliseconds. Following sapanisertib dosing on day 1, the mean QTcI values remained normal, from 408.9 to 422.1 milliseconds. The mean values of ΔQTcI ranged from –8.8 to 7.1 milliseconds (Figure 1A). The estimated LSM changes of ΔQTcI values ranged from –9.1 to 7.1 milliseconds (Table 2). The maximum estimated LSM value of ΔQTcI was 7.1 milliseconds at 24 hours after dosing, with an associated maximum 1‐sided 95% upper confidence bound (UCB) of 11.4 milliseconds. No other values of UCB exceeded 10 milliseconds.

**Table 2:** Mean Changes From Time‐Matched Baseline in QTcI and QTcF

Hours After Sapanisertib Dose	LSM QTcI (msec) Change (95% UCB)	LSM QTcF (msec) Change (95% UCB)
0.25	–2.2 (2.2)	–1.7 (2.2)
0.5	–8.3 (–4.0)	–5.3 (–1.5)
1	–1.8 (2.6)	1.8 (5.6)
1.5	–2.7 (1.7)	0.1 (4.0)
2	–5.0 (–0.6)	–3.7 (0.1)
2.5	–7.6 (–3.2)	–7.4 (–3.6)
3	–9.1 (–4.6)	–7.6 (–3.7)
4	–7.2 (–2.9)	–8.0 (–4.2)
6	–8.8 (–4.6)	–10.1 (–6.4)
8	–2.2 (2.4)	–4.2 (–0.3)
10	–6.6 (–0.5)	–12.5 (–7.1)
24	7.1 (11.4)	0.8 (4.5)
48	2.8 (8.1)	1.2 (5.8)

LSM, least squares mean; msec, milliseconds; QT, measure of the time between the start of the Q wave and the end of the T wave in the electrical cycle of the heart; QTc, rate‐corrected QT interval; QTcI, individual baseline corrected rate‐corrected QT interval; QTcF, rate corrected QT interval with Fridericia correction; UCB, upper confidence bound.

Results of the categorical analysis of the absolute QTcI showed that following sapanisertib treatment, 6 of 32 evaluable patients had QTcI >450 milliseconds, including 1 patient with QTcI between 480 and 500 milliseconds (Table 3); however, 2 of these patients had QTcI >450 milliseconds at baseline. Of the 6 patients who had prolonged QTcI following treatment, 4 patients had a ΔQTcI between 30 and 60 milliseconds, and 1 patient had a ΔQTcI >60 milliseconds at any time point (Table 3).

**Table 3:** Results of the Categorical Analyses for QTcI, QTcF, PR, and QRS

Parameter	Criterion	At Baseline n (%)	After 40 mg Sapanisertib Dose n (%)
QTcI	>450 msec	2 (6.3)	6 (18.8)
	>480 msec	1 (3.1)	1 (3.1)
	>500 msec	0 (0)	0 (0)
	ΔQTcI >30 msec at any time point	N/A	5 (15.6)
	ΔQTcI >60 msec at any time point	N/A	1 (3.1)
QTcF	>450 msec	1 (3.1)	2 (6.3)
	>480 msec	0 (0)	0 (0)
	>500 msec	0 (0)	0 (0)
	ΔQTcF >30 msec at any time point	N/A	2 (6.3)
	ΔQTcF >60 msec at any time point	N/A	0 (0)
PR	>200 msec	0 (0)	1 (3.1)
	ΔPR >25% at any time point	N/A	1 (3.1)
QRS	>110 msec	0 (0)	0 (0)
	ΔQRS >25% at any time point	N/A	0 (0)

ΔQTcF, change from time‐matched baseline in QTcF; ΔQTcI, change from time‐matched baseline in QTcI; msec, milliseconds; N/A, not applicable; PR, interval on ECG between the start of the P wave and the beginning of the QRS complex; QRS, interval on ECG between the start of the Q wave and end of the S wave; QT, measure of the time between the start of the Q wave and the end of the T wave in the electrical cycle of the heart; QTc, rate‐corrected QT interval; QTcI, individual baseline corrected rate‐corrected QT interval; QTcF, rate‐corrected QT interval with Fridericia correction.

For QTcF, mean values were normal and slightly lower than those for QTcI at baseline, from 404.6 to 413.5 milliseconds, and after dosing, from 399.4 to 413.5 milliseconds. The mean ΔQTcF values were between –11.9 and 2.5 milliseconds (Figure 1B). The estimated LSM values of ΔQTcF ranged from –12.5 to 1.8 milliseconds. The maximum LSM value of ΔQTcF at 1 hour after dosing was 1.8 milliseconds with a 1‐sided 95% UCB of 5.6 milliseconds, but the maximum UCB was 5.8 milliseconds at 48 hours after dosing with an LSM of 1.2 milliseconds (Table 2).

Analysis of the absolute QTcF values showed that 2 of 32 evaluable patients had QTcF between 450 and 480 milliseconds following treatment, including 1 patient with a preexisting prolonged QTcF (Table 3). Both patients had ΔQTcI between 30 and 60 milliseconds at any time point.

### Heart Rate Assessment (Repeated‐Measures Mixed‐Effects Analysis Population)

**Figure 2:**
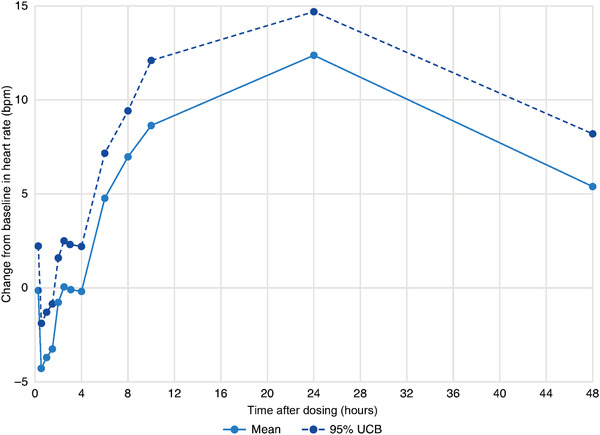
Mean time‐matched changes from baseline in heart rate. bpm, beats per minute; UCB, upper confidence bound.

Mean heart rate values were normal, both at baseline, between 68.1 and 74.0 beats per minute (bpm), and during treatment, between 64.5 and 88.4 bpm. Mean changes ranged from –4.7 to 12.4 bpm. Estimated LSM changes in heart rate from time‐matched baseline were between –4.4 and 12.4 bpm, with the maximum at 24 hours after dosing (Table S1). At 10 hours after dosing, the LSM increase was 8.6 bpm, and the time course of the findings indicated a consistent trend toward greater values with later time points and a slight decrease at 48 hours (Figure 2). Minimal increases in heart rate were observed following administration of sapanisertib 40 mg, with a maximum LSM change from time‐matched baseline of 12.4 bpm. However, mean heart rate values were within the normal range after sapanisertib treatment.

### Pharmacokinetics (PK Analysis Population)

**Figure 3:**
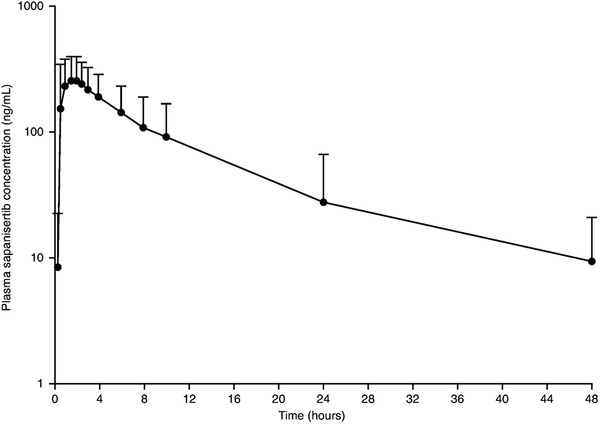
Mean (SD) plasma concentration–time profile of sapanisertib (semilogarithmic scale) following administration of a single dose of 40 mg.^*^ SD, standard deviation. ^*^All sapanisertib plasma concentrations that were below the limit of quantitation were set as zero and included in the calculation of mean values.

A summary of plasma PK parameters is provided in Table S2. Sapanisertib exhibited fast oral absorption following a 40‐mg single oral dose on an empty stomach with a median time to maximum plasma concentration of 1.52 hours (range, 0.5‐24.0), geometric mean C_max_ of 297 ng/mL (%CV, 52.9%) and geometric mean area under the concentration‐time curve from time 0 to infinity of 2480 ng • h/mL (%CV, 80.4%). Geometric mean apparent oral clearance was 16.1 L/h (%CV, 46.3%). Sapanisertib concentrations declined with a mean plasma half‐life of 9.5 hours (standard deviation, 2.9 hours). The mean concentration‐time profile of sapanisertib is shown in Figure 3.

### Relationship of Sapanisertib Concentration and QTc (Concentration‐Effect Analysis Population)

**Figure 4:**
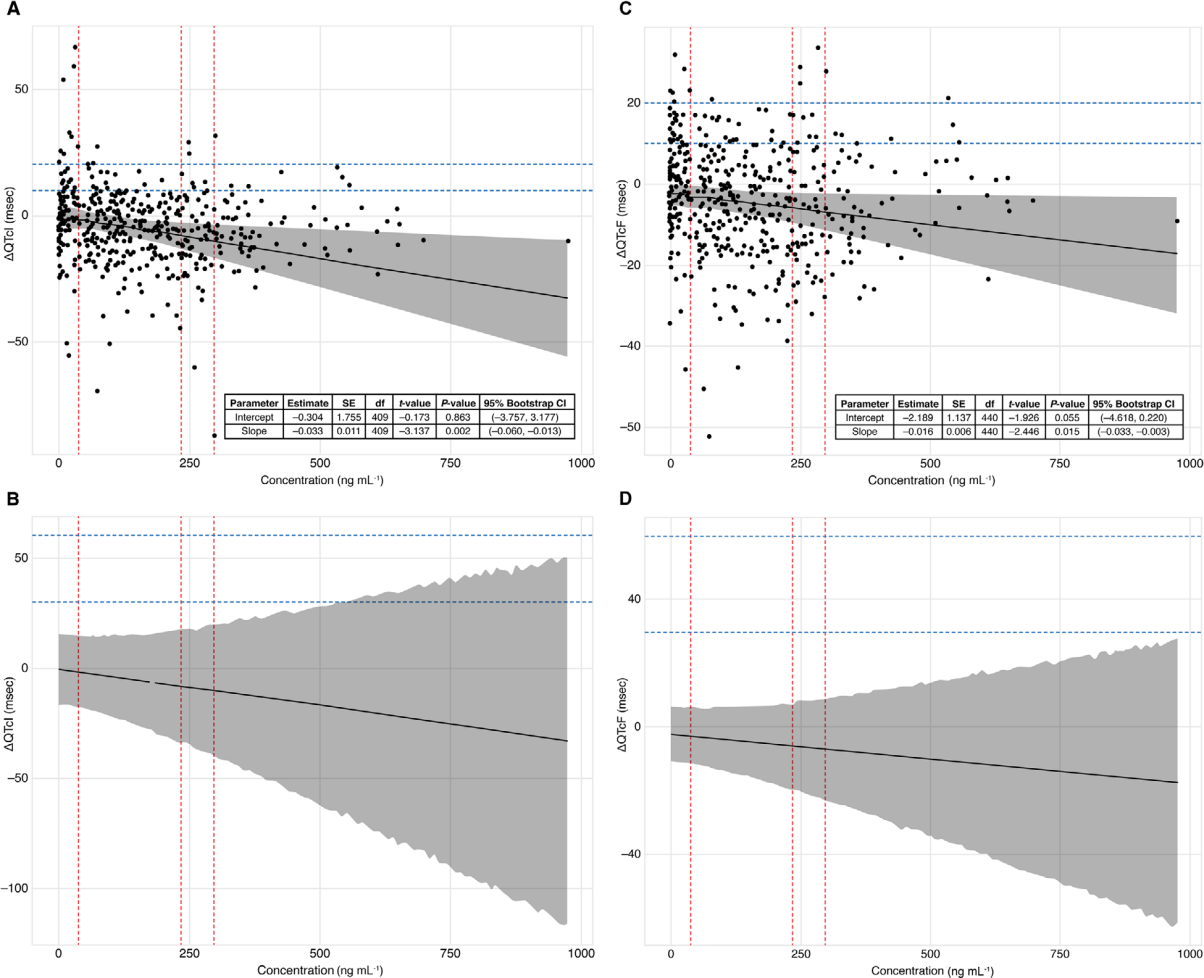
Relationship of ΔQTcI and ΔQTcF with plasma concentration of sapanisertib. (A) Plot of the linear mixed‐effects model of ΔQTcI vs sapanisertib plasma concentration. Dots represent differences in average QT interval from time‐matched baseline for individual patients at each time point corrected by regression analysis. Solid line and shaded region represent the fitted linear model and associated 95% confidence band, respectively. Dashed vertical reference lines denote the estimated maximum concentration for 4‐, 30‐, and 40‐mg doses of sapanisertib. Dashed horizontal reference lines indicate 10‐ and 20‐msec increases in QTcI. (B) Model‐predicted ΔQTcI as a function of sapanisertib plasma concentration with associated 90% prediction interval. Solid line represents estimated population mean function for ΔQTcI as a function of sapanisertib plasma concentration based on the fitted linear effects model for ΔQTcI, with the 90% prediction interval shaded in gray. Dashed vertical reference lines denote the estimated maximum concentration for 4‐, 30‐, and 40‐mg doses of sapanisertib. Dashed horizontal reference lines indicate 30‐ and 60‐msec increases in QTcI. (C) Plot of the linear mixed‐effects model of ΔQTcF vs sapanisertib plasma concentration. Dots represent differences in average QT interval from time‐matched baseline for individual patients at each time point corrected by regression analysis. Solid line and shaded region represent the fitted linear model and its associated 95% confidence band, respectively. Dashed vertical reference lines denote the estimated maximum concentration for 4‐, 30‐, and 40‐mg doses of sapanisertib. Dashed horizontal reference lines indicate 10‐ and 20‐msec increases in QTcF. (D) Model‐predicted ΔQTcF as a function of sapanisertib plasma concentration with associated 90% prediction interval. Solid line represents estimated population mean function for ΔQTcF as a function of sapanisertib plasma concentration based on the fitted linear effects model for ΔQTcI, with the 90% prediction interval shaded in gray. Dashed vertical reference lines denote the estimated maximum concentration for 4‐, 30‐, and 40‐mg doses of sapanisertib. Dashed horizontal reference lines indicate 30‐ and 60‐msec increases in QTcF. CI, confidence interval; df, degrees of freedom; msec, milliseconds; ΔQTcF, change from time‐matched baseline in QTcF; ΔQTcI, change from time‐matched baseline in QTcI; QT, measure of the time between the start of the Q wave and the end of the T wave in the electrical cycle of the heart; QTc, rate‐corrected QT interval; QTcF, rate‐corrected QT interval with Fridericia correction; QTcI, individual baseline corrected rate‐corrected QT interval; SE, standard error.

A linear mixed‐effects model was used to characterize the direct effect of sapanisertib plasma concentration on ΔQTcI. Based on this model, ΔQTcI had a weakly negative association with sapanisertib plasma concentration (Figure 4A). The linear mixed‐effects model of sapanisertib plasma concentration versus ΔQTcI was subsequently used to predict the effect of sapanisertib on QTcI at the expected C_max_ values for 4‐, 30‐, and 40‐mg sapanisertib doses. C_max_ values of 36.9 and 235.0 ng/mL, which corresponded with 4‐ and 30‐mg sapanisertib, respectively, were from prior sapanisertib studies in which patients with advanced solid tumors were treated with single‐agent sapanisertib at 4 mg once daily or 30 mg once weekly (data on file^14^) (Table 4) in fasted state as immediate release capsules. C_max_ value of 297.0 ng/mL for sapanisertib 40 mg was established from the current study for sapanisertib also administered as immediate‐release capsules. Model‐based simulations from the fitted model for ΔQTcI versus sapanisertib plasma concentration were used to generate CIs across the range of observed concentrations and to estimate the probability of patient ΔQTcI values exceeding 30‐ and 60‐millisecond thresholds at C_max_ values corresponding to the 4‐, 30‐, and 40‐mg doses (Table 4). Based on this model, the upper % confidence limit of the 2‐sided 95%CI, ΔQTcI did not exceed 10 milliseconds at the maximum concentrations corresponding to the 4‐, 30‐, and 40‐mg sapanisertib doses (Table 4 and Figure 4A). Specifically, the estimated probability that ΔQTc would exceed 30 milliseconds was ≤0.0139, and the estimated probability that ΔQTc would exceed 60 milliseconds was ≤0.0001 at any of the specified sapanisertib doses, up to and including 40 mg (Table 4).

**Table 4:** Model‐Based Estimates of ΔQTcI and ΔQTcF at C_max_ for Sapanisertib Doses of 4, 30, and 40 mg

			Estimated ΔQTc (msec)		Estimated Probability	
Sapanisertib Dose	Geometric Mean C_max_	Model	Estimate	95% Upper Confidence Limit	ΔQTc >30 msec	ΔQTc >60 msec
4 mg	36.9 ng/mL^a^	ΔQTcF	–2.762	–0.881	<0.0001	<0.0001
		ΔQTcI	–1.538	1.432	0.0006	<0.0001
30 mg	235 ng/mL^b^	ΔQTcF	–5.837	–2.869	<0.0001	<0.0001
		ΔQTcI	–8.159	–3.673	0.0050	<0.0001
40 mg	297 ng/Ml^c^	ΔQTcF	–6.800	–3.374	<0.0001	<0.0001
		ΔQTcI	–10.231	–4.990	0.0139	0.0001

C_max_, single‐dose maximum (peak) concentration; msec, milliseconds; ΔQTcF, change from time‐matched baseline in QTcF; ΔQTcI, change from time‐matched baseline in QTcI; QT, measure of the time between the start of the Q wave and the end of the T wave in the electrical cycle of the heart; QTc, rate‐corrected QT interval; QTcI, individual baseline corrected rate‐corrected QT interval; QTcF, rate‐corrected QT interval with Fridericia correction.

a Observed C_max_ for 4‐mg dose of sapanisertib administered fasted in the once‐daily dosing schedule. No meaningful plasma accumulation of sapanisertib was observed with repeat once‐daily dosing.

b Observed C_max_ for a 30‐mg dose of sapanisertib administered fasted in the once‐weekly dosing schedule.

c Data from current study.

A linear mixed‐effects model was used to characterize the direct effect of sapanisertib concentration on the ΔQTcF and showed a weakly negative association between sapanisertib plasma concentration and ΔQTcF (Figure 4C). Using model‐based simulations from a fitted linear mixed‐effects model for ΔQTcF vs sapanisertib plasma concentration, the 95% confidence limits for ΔQTcF were shown to not exceed 10 milliseconds at the peak concentrations corresponding to the 4‐, 30‐, and 40‐mg sapanisertib doses (Table 4 and Figure 4C). Figure 5B shows the model‐predicted ΔQTcF and associated 90% prediction interval estimated by simulation from the final concentration‐ΔQTcF model. Specifically, the estimated probability that ΔQTcF would exceed 30 milliseconds or 60 milliseconds was <0.0001 at any of the specified sapanisertib doses (Table 4).

### Relationship of Sapanisertib Concentration and Heart Rate (Concentration‐Effect Analysis Population)

A linear mixed‐effects model was used to characterize the direct effect of sapanisertib concentration on the change from time‐matched baseline in RR (ΔRR), in which ΔRR had a weakly positive association with the sapanisertib plasma concentration (Figure S2).

### Safety (Safety Population)

A total of 43 (98%) patients experienced adverse events (AEs) during the study. The most common any‐grade AEs, regardless of causality, included nausea (n = 35; 80%), fatigue (n  =  27; 61%), and vomiting (n = 25; 57%) (Table S3). Overall, 26 (59%) patients reported grade ≥3 AEs, the most common were fatigue (n = 7; 16%), diarrhea, stomatitis, hyperglycemia, and dehydration (n = 2; 5% each) (Table S3). The rates of AEs of special interest included hyperglycemia (n = 8; 18%), rash (n = 9; 20%), renal insufficiency (n = 2; 5%), mucosal inflammation (n = 11; 25%), and asthenic conditions (n = 27; 61%). Four patients discontinued study due to AEs of grade 4 malignant neoplasm and pelvic pain, grade 3 breast cancer, grade 2 eye pain, and grade 2 decreased weight, with eye pain and decreased weight considered as drug related. Two patients died during the study: 1 patient due to metastatic breast cancer on cycle 1 day 5 and a second patient due to a small intestinal perforation on cycle 1 day 37. Neither of these deaths were considered by the investigator to be related to the study drug.

## Discussion

The primary objective of this DQT study was to characterize the effect of a single dose of sapanisertib 40 mg on the QTc interval in patients with advanced solid tumors. The change from time‐matched baseline in QTcI was evaluated using both a traditional statistical analysis and by a quantitative evaluation of the concentration‐QTc relationship. The 40‐mg single dose was the highest dose of sapanisertib evaluated clinically in a once‐weekly schedule^15^ and was expected to produce a total plasma C_max_ of ∼297 ng/mL, which would exceed the C_max_ of sapanisertib from a dose of 4 mg (∼37 ng/mL) or 30 mg (∼235 ng/mL) sapanisertib. This supratherapeutic dose allowed for assessment of the effect of sapanisertib on the QTc interval at concentrations that were higher than those expected at the clinical doses of 4 mg once daily or 30 mg once weekly, the recommended phase 2 dose of sapanisertib in their respective dosing schedules. Furthermore, time‐matched collection of serial PK data of sapanisertib for concentration‐QTc analysis enabled the quantitative understanding of the relationship between plasma sapanisertib concentrations and any changes in QTc for projection of the effect of sapanisertib at doses that were not evaluated in this study.

ECG analysis showed normal QTcI and QTcF values at baseline and after a single sapanisertib 40 mg dose. Based on the results of the primary statistical analysis, following administration of sapanisertib 40 mg, the maximum LSM ΔQTcI was 7.1 milliseconds with an associated upper limit of the 1‐sided 95%CI of 11.4 milliseconds at a single time point of 24 hours after dosing. For all other time points, the upper bound of the 1‐sided 95%CI for change in QTcI was below 10 milliseconds. This LSM change from time‐matched baseline and the upper limit of the 1‐sided 95%CI exceeded the 5‐ and 10‐millisecond ICH E14 thresholds, respectively. However, it is important to emphasize that these thresholds apply to TQT studies, which typically include placebo and positive control arms and are conducted in healthy volunteers, to exclude small effects (ie, <10‐millisecond increase) on QTc. Therefore, although the upper limit of the 1‐sided 95%CI in this study slightly exceeded the 10‐millisecond ICH E14 threshold, it was nonetheless well below the 20‐millisecond threshold considered to be applicable and clinically relevant to the interpretation of results of DQT studies investigating anticancer agents.^5^ For QTcF, the maximum LSM change from time‐matched baseline was 1.8 milliseconds (ie, <5‐millisecond ICH E14 threshold for mean effect), with an upper limit of the 1‐sided 95%CI of 5.6 milliseconds, which was below the 10‐millisecond ICH E14 threshold.

Importantly, evidence of the lack of QT prolongation effect of sapanisertib was demonstrated by the exposure‐response (PK‐QTc) modeling, which used the PK time‐matched ECG data to describe the relationship between sapanisertib plasma concentration and change from time‐matched baseline QTcI/QTcF. Concentration‐QT analysis is widely recognized as a robust approach to quantitatively evaluate the effects of an investigational agent on QT interval, particularly in the oncology therapeutic area.^4^, ^6^, ^16^, ^17^, ^18^, ^19^, ^20^, ^21^ The results from this analysis indicate that there were no concentration‐dependent increases in ΔQTcI or ΔQTcF. This PK‐QTc model was also used to predict the distribution of ΔQTc over the range of sapanisertib concentrations and particularly at the expected C_max_ for sapanisertib doses of 4, 30, and 40 mg. At the expected C_max_ for these doses, all point estimates for ΔQTc were negative, with 95% upper confidence limits <10 milliseconds. Similarly, estimates for the probability that ΔQTc would exceed 30 milliseconds were not >0.0139, and estimates for the probability that ΔQTc would exceed 60 milliseconds were not >0.0001 for sapanisertib doses of 4, 30, and 40 mg.

Overall, the administration of sapanisertib (40 mg single dose and 30 mg once weekly) was well tolerated. The safety profile was consistent with previous studies investigating single‐agent sapanisertib at varying dosages and schedules.^14^, ^22^ Furthermore, the AEs reported in this study were consistent with other mTOR inhibitors,^23^, ^24^, ^25^, ^26^ with no new safety concerns identified.

## Conclusions

Taken together, these results support the conclusion that sapanisertib 40 mg does not produce clinically relevant (ie, >20 milliseconds) effects on the QT interval in patients with advanced solid tumors and therefore support the ongoing clinical investigation of sapanisertib. Importantly, there was no concentration‐dependent increase in the change from time‐matched baseline QTcI or QTcF in the PK/QTc analysis.

## Conflicts of Interest

C.P., L.R., and K.V. are employees of Millennium Pharmaceuticals, Inc., Cambridge, Massachusetts, a wholly owned subsidiary of Takeda Pharmaceutical Company Limited; C.P. owns stocks of with Takeda Pharmaceuticals International Co.; S.G. received research funding for this trial from Millennium Pharmaceuticals, Inc.; J.W. serves as a paid consultant for this work for Millennium Pharmaceuticals, Inc.; Y.S. was an employee of Millennium Pharmaceuticals, Inc., at the time of this work and owns stocks with Takeda Pharmaceuticals International Co.; and M.P. and A.C.L. declare no conflicts of interest.

## Funding

This work was supported by funding from Millennium Pharmaceuticals, Inc., Cambridge, Massachusetts, a wholly owned subsidiary of Takeda Pharmaceutical Company Limited.

## Author Contributions

All authors contributed to the writing and critically revised the manuscript, reviewed and approved the final version of the manuscript, and agreed to be accountable for all aspects of the work.

## Data Sharing

Takeda makes patient‐level, deidentified data sets and associated documents available after applicable marketing approvals and commercial availability have been received, an opportunity for the primary publication of the research has been allowed, and other criteria have been met as set forth in Takeda's Data Sharing Policy (see https://www.takedaclinicaltrials.com/ for details). To obtain access, researchers must submit a legitimate academic research proposal for adjudication by an independent review panel, who will review the scientific merit of the research and the requestor's qualifications and conflict of interest that can result in potential bias. Once approved, qualified researchers who sign a data‐sharing agreement are provided access to these data in a secure research environment.

## Supporting information


**Table S1**. Mean Changes From Time‐Matched Baseline in Heart Rate
**Table S2**. Summary of Plasma PK Parameters
**Table S3**. Most Common Any‐Grade and Grade ≥3 Adverse Events Occurring in ≥10% of Patients
**Figure S1**. Regression of QT (uncorrected) vs RR interval
Regression of QTcI vs RR interval
Regression of QTcF vs RR interval
**Figure S2**. Relationship of ΔRR and plasma concentration of sapanisertib. Plot of the linear mixed‐effects model of ΔRR vs sapanisertib plasma concentration. Dots represent differences in average RR interval from time‐matched baseline for individual patients at each time point. The solid line is the fitted linear model and the shaded region is its associated 95% confidence band.
