# Supplementary material for: Phase 1 Study to Evaluate the Effect of the Investigational Anticancer Agent Sapanisertib on the QTc Interval in Patients With Advanced Solid Tumors
Source: Clin Pharmacol Drug Dev. 2020 Jun 2;9(7):876–88. doi: 10.1002/cpdd.808 (PMC7586797; doi:10.1002/cpdd.808)
Supplement: Supplementary file 1 — Table S1. Mean Changes From Time‐Matched Baseline in Heart Rate Table S2. Summary of Plasma PK Parameters Table S3. Most Common Any‐Grade and Grade ≥3 Adverse Events Occurring in ≥10% of Patients Figure S1. Regression of QT (uncorrected) vs RR interval Regression of QTcI vs RR interval Regression of QTcF vs RR interval Figure S2. Relationship of ΔRR and plasma concentration of sapanisertib. Plot of the linear mixed‐effects model of ΔRR vs sapanisertib plasma concentration. Dots represent differences in average RR interval from time‐matched baseline for individual patients at each time point. The solid line is the fitted linear model and the shaded region is its associated 95% confidence band. [file CPDD-9-876-s001.docx]

**Phase 1 Study to Evaluate the Effect of the Investigational Anti-Cancer Agent Sapanisertib on the QTc Interval in Patients with Advanced Solid Tumors**

Chirag Patel, Sanjay Goel, Manish Patel, Lakshmi Rangachari, Jayson D. Wilbur, Yaping Shou, Karthik Venkatakrishnan, and A. Craig Lockhart

**Supplemental Methods**

*Patients*

Eligible patients were 18 years or older, with a radiographically or clinically evaluable untreated solid tumor (but did not have to meet the measurable disease guidelines set in the modified Response Evaluation Criteria in Solid Tumours, version 1.1) and had an ECOG performance status of 0 or 1. Patients were also required to have a left ventricular ejection within five absolute percentage points of institutional standard of normal within 28 days prior to the first dose of study drug; adequate hepatic, renal, and hematologic functions; and fasting glucose of ≤120 mg/dL and fasting triglycerides of ≤300 mg/dL. Patients were excluded if they had received treatment with any investigational products within 14 days or systemic anti-cancer therapy within 28 days of the first dose of study drug. Other treatments that were not permitted included the use of systemic corticosteroid, proton pump inhibitors, or moderate CYP2C19 inhibitors within 7 days of the first dose of study drug; or strong CYP3A4 and CYP2C19 inducers and/or inhibitors or major surgery within 14 days of the first dose of study drug. Patients who had gastrointestinal disease, diabetes mellitus, tumors with involvement of the mediastinum, or significant active cardiovascular (including baseline QTcF >430 msec for men QTcF >450 msec for females or congenital long QT syndrome) or pulmonary disease at study entry were also excluded.

**Supplementary Table S1**. Mean Changes From Time-Matched Baseline in Heart Rate

| Hours after sapanisertib dose | LSM heart rate (bpm) change (95% UCB) |
| --- | --- |
| 0.25 | –0.2 (2.2) |
| 0.5 | –4.4 (–1.9) |
| 1 | –3.8 (–1.4) |
| 1.5 | –3.3 (–0.9) |
| 2 | –0.8 (1.5) |
| 2.5 | 0.1 (2.5) |
| 3 | –0.1 (2.3) |
| 4 | –0.2 (2.2) |
| 6 | 4.8 (7.1) |
| 8 | 6.9 (9.4) |
| 10 | 8.6 (12.1) |
| 24 | 12.4 (14.7) |
| 48 | 5.4 (8.3) |

bpm, beats per minute; LSM, least squares mean; UCB, upper confidence bound.

**Supplementary Table S2**. Summary of plasma PK parameters

| Parameter | Mean (SD) | Geometric mean (%CV) | Median (range) |
| --- | --- | --- | --- |
| C_max_ (ng/mL) | 337 (178) | 297 (52.9) | 317 (94–976) |
| T_max_ (h) | 2.51 (3.65) | 1.73 (145.7) | 1.52 (0.50–24.00) |
| CL (L/h) | 18.3 (8.5) | 16.1 (46.3) | 18.0 (3.1–41.8) |
| AUC_∞_ (ng*h/mL) | 2993 (2405) | 2480 (80.4) | 2220 (958–12944) |
| T_1/2_ | 9.48 (2.85) | 9.06 (30.1) | 9.07 (4.48–16.87) |

AUC_∞_, area under the plasma concentration–time curve from zero to infinity; CL, apparent clearance; C_max_, single-dose maximum (peak) concentration; PK, pharmacokinetic; t_1/2_, terminal disposition half-life; T_max_ single-dose time to maximum (peak) concentration.

**Supplementary Table S3**. Most Common Any-Grade and Grade ≥3 Adverse Events Occurring in ≥10% of Patients

|  | Total N = 44 | |
| --- | --- | --- |
| n (%) | Any grade | Grade ≥3 |
| Nausea | 35 (80) | 1 (2) |
| Fatigue | 27 (61) | 7 (16) |
| Vomiting | 25 (57) | 1 (2) |
| Decreased appetite | 20 (45) | 1 (2) |
| Diarrhea | 15 (34) | 2 (5) |
| Weight decreased | 14 (32) | 1 (2) |
| Urinary tract infection | 12 (27) | — |
| Stomatitis | 10 (23) | 2 (5) |
| Constipation | 9 (20) | — |
| Cough | 9 (20) | — |
| Dizziness | 9 (20) | — |
| Headache | 8 (18) | — |
| Hyperglycemia | 8 (18) | 2 (5) |
| Back pain | 7 (16) | 1 (2) |
| Dyspnea | 7 (16) | — |
| Rash maculopapular | 7 (16) | 1 (2) |
| Dehydration | 6 (14) | 2 (5) |
| Depression | 6 (14) | — |
| Dry mouth | 5 (11) | — |
| Dysgeusia | 5 (11) | — |
| Dyspepsia | 5 (11) | — |
| Insomnia | 5 (11) | — |

**Supplementary Figure S1.**

1. Regression of QT (uncorrected) versus RR interval


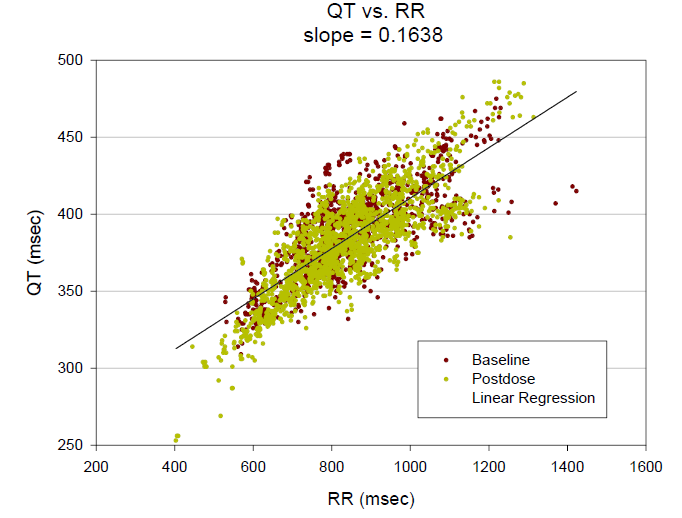


1. Regression of QTcI versus RR interval


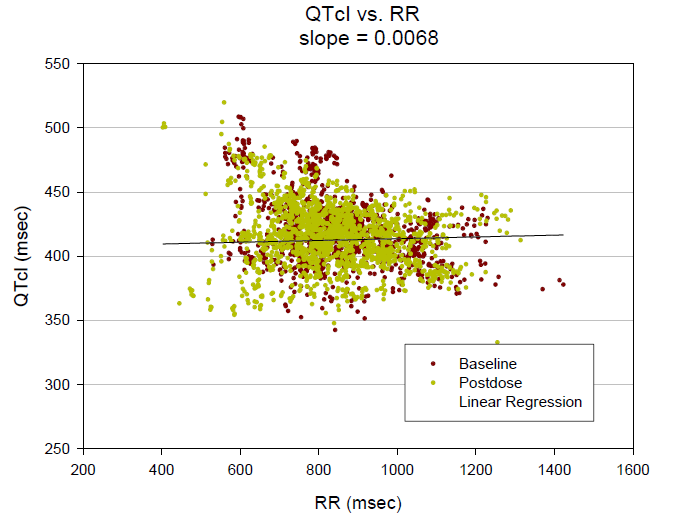


1. Regression of QTcF versus RR interval


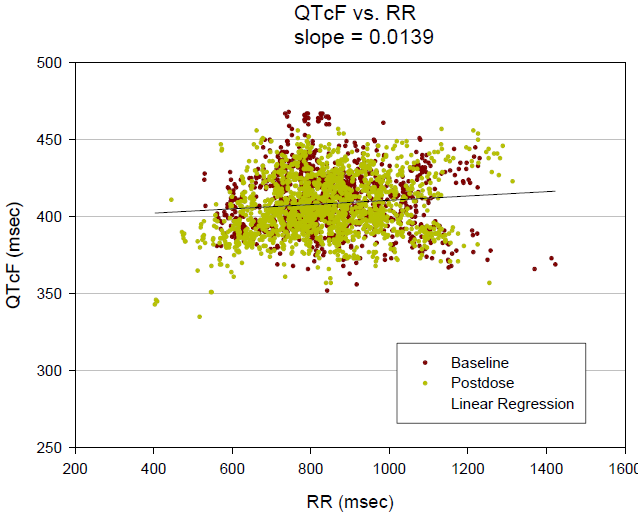


**Supplementary Figure S2.** Relationship of ΔRR and plasma concentration of sapanisertib. Plot of the linear mixed-effects model of ΔRR versus sapanisertib plasma concentration. Dots represent differences in average RR interval from time-matched baseline for individual patients at each time point. The solid line is the fitted linear model and the shaded region is its associated 95% confidence band.


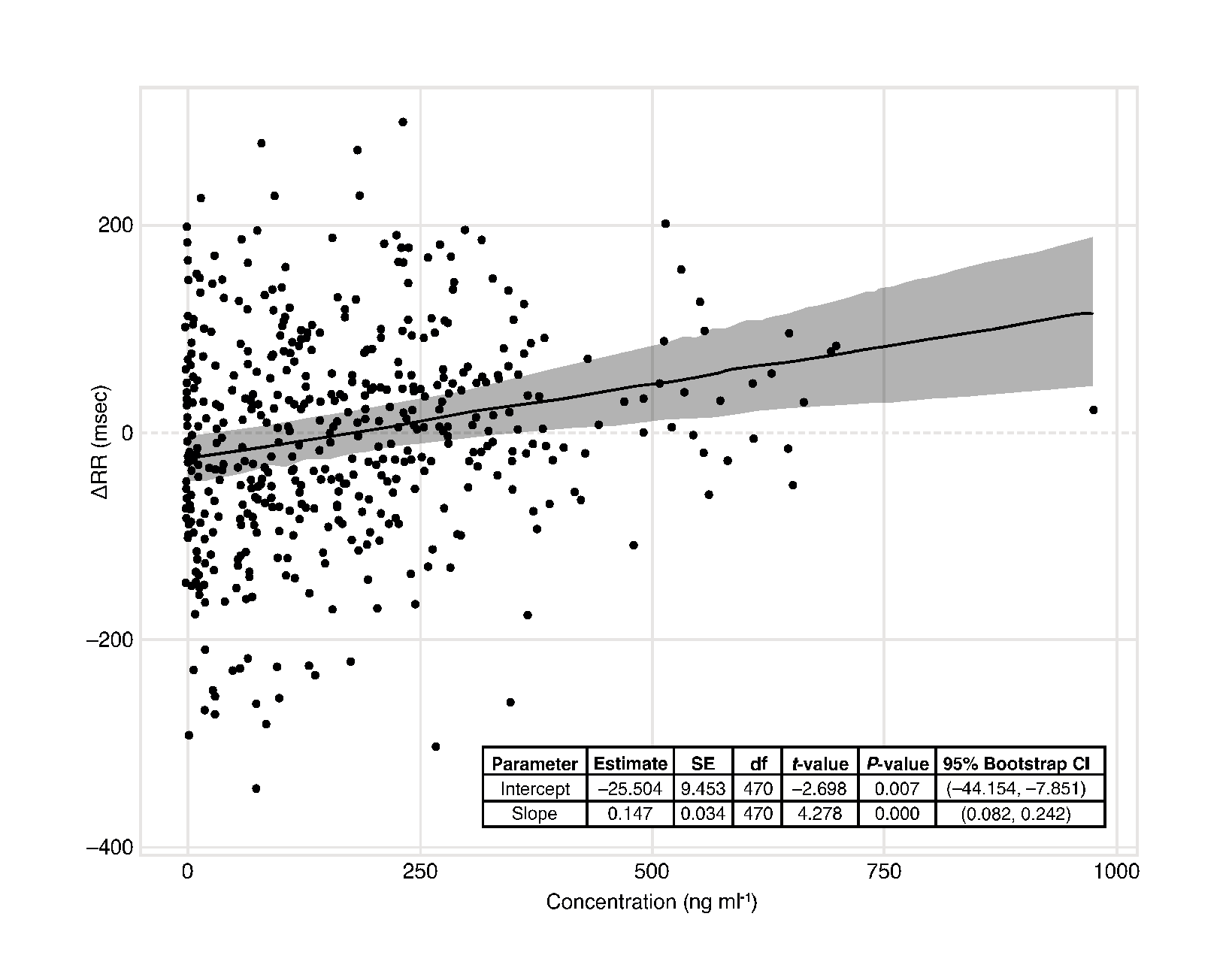


CI, confidence interval; df, degrees of freedom; ΔRR, change from time-matched baseline in time elapsing between 2 consecutive R waves in an Electrocardiogram. SE, standard error.
